# Gray Matter Abnormalities in the Inhibitory Circuitry of Young Binge Drinkers: A Voxel-Based Morphometry Study

**DOI:** 10.3389/fpsyg.2017.01567

**Published:** 2017-09-13

**Authors:** Sónia S. Sousa, Adriana Sampaio, Paulo Marques, Óscar F. Gonçalves, Alberto Crego

**Affiliations:** ^1^Neuropsychophysiology Lab, CIPsi, School of Psychology, University of Minho Braga, Portugal; ^2^Life and Health Sciences Research Institute, School of Health Sciences, University of Minho Braga, Portugal; ^3^ICVS/3B’s – PT Government Associate Laboratory Guimarães, Portugal; ^4^Spaulding Neuromodulation Center, Department of Physical Medicine and Rehabilitation, Spaulding Rehabilitation Hospital and Massachusetts General Hospital, Harvard Medical School, Charlestown MA, United States; ^5^Department of Applied Psychology, Bouvé College of Health Sciences, Northeastern University, Boston MA, United States

**Keywords:** binge drinking, gray matter, inhibitory control, self-regulation, impulsivity, adolescence, college-students, voxel-based morphometry

## Abstract

Binge drinking (BD) is defined as a pattern of high alcohol intake in a short time followed by periods of abstinence. This behavior is very common in adolescence, a developmental stage characterized by the maturation of the prefrontal and striatal networks, important circuits underlying the capacity to control and regulate the behavior. In this study, we conducted a voxel-based morphometry (VBM) analysis, using a region of interest (ROI) analysis of brain regions associated with inhibitory control and self-regulatory processes, in a group of 36 young college students, 20 binge drinkers (BDs) and 16 alcohol abstinent controls (AAC). Results showed increased gray matter (GM) densities in the left middle frontal gyrus in BDs, when compared with alcohol abstinent controls. Additionally, a ROI-based Pearson analysis documented positive correlations between the left middle frontal gyrus GM densities and the self-control subscale of the Barratt Impulsiveness Scale (BIS), in the BD group. These findings highlight abnormalities in core brain regions associated with self-regulatory processes in the BD group.

## Introduction

Binge drinking (BD) is a common pattern of consumption among college students and is characterized by repeated episodes of large amounts of alcohol intake. In order to be considered BD, an alcohol ingestion episode requires a minimum consumption of four drinks for women and five for men, in a brief period of time (±2 h) at least once per month, being followed by periods of abstinence (see Parada et al., 2011; Substance Abuse and Mental Health Services Administration [SAMHSA], 2016a for a review). This pattern of high alcohol consumption increases the individual’s susceptibility to engage in several risky behaviors (Courtney and Polich, 2009; Substance Abuse and Mental Health Services Administration [SAMHSA], 2015).

According to the National Institute on Alcohol Abuse and Alcoholism (NIAAA), nearly 60% of US college students (age range 18–22 years) reported alcohol consumption and 40% exhibited a BD pattern in the past month. This behavior is seriously harmful affecting several domains of the individuals’ life such as social, academic and health, being associated with the death of approximately 1,825 US college students each year (National Institute on Alcohol Abuse and Alcoholism [NIAAA], 2015; Substance Abuse and Mental Health Services Administration [SAMHSA], 2016b). In Europe, a growth of this abusive pattern of consumption among young people was noted between the years 1995 and 2000, with the prevalence rate being quite unchangeable over the past two decades (Kraus et al., 2016). The percentage of frequent BD (at least a BD episode per week) is higher in the youngest individuals (age range: 15–24 years) with 33% reporting BD. Additionally, in terms of gender prevalence, the proportion of frequent BD is higher in men (36%) than in women (19%). (Eurobarometer, 2010).

The adolescence is a critical period for the beginning of abusive alcohol consumption (Crews and Boettiger, 2009; Casey and Caudle, 2013). In fact the onset of BD among youths seems to be around age 12, but the largest percentage of BD episodes is observed in older adolescents (age range: 16–17) (National Institute on Alcohol Abuse and Alcoholism [NIAAA], 2015). In this particular developmental period individuals tend to increasingly engage in social behaviors, such as recreational drinking, in order to attain social conformity. Additionally, adolescents tend to use alcohol as a coping strategy to deal with negative emotions and achieving an illusive state of well-being, induced by large doses of alcohol (Lorant et al., 2013; Laghi et al., 2016). At the neuromaturational level, adolescence is a period of great physiological changes including intracellular events such as loss of overproduced synapses and increase of myelin sheaths, particularly in the prefrontal cortex, limbic system, and white matter association and projection fibers, essential to brain maturation. In addition, these neuromaturational changes are linked with advancements in complex cognitive functions as inhibitory control and self-regulation, which also occur in this developmental stage (Bava et al., 2010), allowing individuals to deal with risk-taking choices (Casey and Caudle, 2013). In accordance, the lower adolescent’s proficiency in regulating their own behavior and suppress inappropriate emotions or actions has been associated with the structural immaturity of several cortical and subcortical regions (Crews and Boettiger, 2009; Bava and Tapert, 2010; Bari and Robbins, 2013), hence, the immature prefrontal-striatal regions in association with the underdeveloped self-regulation seem to let individuals more prone to risky environmental factors such as drugs or alcohol misuse, and to engage in impulsive behaviors.

Research on the putative neuropsychological abnormalities in BD documented a multiplicity of deficits underlying these executive function and self-regulatory abilities, as attention, cognitive flexibility, working memory, planning, decision-making and inhibitory control. These functional impairments have been associated with atypical functioning of several brain regions, including the dorsolateral prefrontal cortex (DLPFC), the inferior, middle and superior frontal gyri, the anterior cingulate cortex and the parietal and the temporal lobes, revealed by electrophysiological, structural and functional neuroimaging studies (see Hermens et al., 2013 and Lopez-Caneda et al., 2014 for a review). In particular, morphometric studies reported regions of enlarged gray matter (GM) such as the striatum (age range: 18–28; Howell et al., 2013), the DLPFC (age range: 20–24; Doallo et al., 2014), the cingulate cortex and the temporal gyri (age range: 22–28; Heikkinen et al., 2017), while some studies showed decreased GM in the temporal gyri, the superior and middle frontal gyri and the pars triangularis (age range: 14–19) (Luciana, et al., 2013; Wilson, et al., 2015).

Overall, while some of the authors related their results to a neurotoxic effect of alcohol (e.g., Luciana et al., 2013; Doallo et al., 2014), others suggested that premorbid changes in the brain structure were present before alcohol initiation, which were possibly related with future alcohol misuse (Cheetham et al., 2014, 4 year follow-up; Squeglia et al., 2014, 2017, 3 year follow-up; Wilson et al., 2015, 1 year follow-up). Therefore, the data gathered in the BD field suggests that the brain abnormalities found in the young BDs might be related with disruptions in the normative brain development that occur previously to the drinking onset, whereas others suggest that alteration of the optimal brain maturation and integrity is a consequence of BD.

Taking this developmental, neurofunctional and neurocognitive findings into consideration, we hypothesized that BDs would show morphological alterations within core brain regions associated with self-regulatory processes (i.e., superior, middle and inferior frontal gyri, orbitofrontal cortex, anterior cingulate, nucleus accumbens and caudate), when compared with AAD. In order to test this hypothesis, we performed a voxel-based morphometry (VBM) study in a group of young college students that met the criteria for binge alcohol consumption and a group of AACs employing a region of interest (ROI) analysis of brain regions associated with inhibitory control and self-regulatory processes (Crews and Boettiger, 2009; Koob and Volkow, 2010).

## Materials and Methods

### Participants

Participants were recruited through an online survey with college students, which included items regarding the use of alcohol (frequency of alcohol consumption, number of drinks consumed on each day of the past week, speed of drinking, etc.) and other drugs (type of drug, frequency of consumption, etc.). Then, participants with BD or AAC criteria were selected to participate and invited to a clinical structured interview. The interview covered several aspects related to alcohol and drug consumption, personal and family history of alcoholism, medical or psychopathological disorders, as well as the assessment of their laterality, impulsivity and psychopathological symptomatology. The sample included 36-college students ranging in age between 18 and 23 years old, with 20 participants with BDs (10 women) and 16 AACs (10 women). Participants were classified as BDs if they consumed a minimum of four drinks or five for men in a brief period of time (∼2 h), at least once per month, for the last 10 months (minimum). Participants assigned to the AAC group were completely alcohol abstinent, i.e., do not drink alcohol at all, neither now nor in the past. The demographic and drinking characteristics of both groups are shown in **Table 1**.

**Table 1:** Demographic and behavioral data for BDs and AACs.

	BD Mean ± SD	AAC Mean ± SD	*t* (34)
N (females)	20 (10)	16 (10)	
Caucasian ethnicity (%)	100	100	
Age (range)	20.45 ± 1.60 (18–23)	21.00 ± 1.71 (18–23)	0.99
Age of onset of BD	17.45 ± 1.08	—	
AUDIT (total score)	11.20 ± 3.25	0.62 ± 1.20	–13.43***
Number of times of BD per month	3.57 ± 1.87	0	–8.54***
Number of months with BD pattern	35.90 ± 14.03	0	–11.44***
Grams of alcohol consumed per week	151 ± 44.27	0	–14.78***
Speed of drinking (gr/h during BD episodes)	34.50 ± 8.26	0	–18.69***
Percentage of times getting drunk when drinking	43.25 ± 20.41	0	–9.48***
Tobacco smokers	7 (4 ♀)	0	
Occasional users of cannabis	2 (2 ♀)	0	
BIS (Total Score)	64.80 ± 5.83	63.56 ± 6.05	–0.62
BIS (Attention)	10.65 ± 2.23	10.63 ± 2.16	–0.03
BIS (Cognitive Instability)	6.10 ± 1.41	5.50 ± 1.51	–1.23
BIS (Motor)	11.80 ± 2.76	10.00 ± 2.03	–2.17*
BIS (Perseverance)	7.05 ± 1.05	7.31 ± 1.40	0.64
BIS (Self-control)	16.50 ± 2.46	17.38 ± 2.58	1.04
BIS (Cognitive complexity)	12.70 ± 1.95	12.75 ± 1.88	0.08

AUDIT, Alcohol Use Disorders Identification Test; BIS, Barratt Impulsiveness Scale; BD, Binge Drinking; AAC, alcohol-abstinent control; SD, standard deviation. All *p*-values reported are for two-tailed independent samples *t*-tests. *P ≤ 0.05. ***P ≤ 0.000.

Exclusion criteria for both groups were defined as the following: be left-handed; scores ≥ 20 in the AUDIT; GSI ≥ 90 or scoring in at least 2 symptomatic dimensions of the SCL-90-R; uncorrected sensory deficits; personal history of traumatic brain injury or neurological disorder; regular (i.e., on a weekly basis) consumption of cannabis, personal history of regular or occasional use of other drugs (opiates, hallucinogens, cocaine, ecstasy, amphetamine compounds or medically prescribed psychoactive substances); Alcohol Use Disorder (AUD), i.e., alcohol abuse/dependence, based on DSM-IV-R criteria; personal and/or family history of any neurological or DSM-IV axis I disorder in first-degree relatives, family history of alcoholism in first-degree relatives; and magnetic resonance imaging (MRI) contraindications.

### Clinical and Neuropsychological Assessment

Personal and family history of alcoholism plus medical or psychopathological disorders information was collected through a semi-structured interview including: a Semi-Structured Assessment for the Genetics of Alcoholism (SSAGA), Individual Assessment Module (IAM) and Family History Assessment Module (FHAM), designed by the Collaborative Study on the Genetics of Alcoholism (Bucholz et al., 1994). In addition, in order to assess the psychopathological symptomatology, the Portuguese version of the Symptom Checklist-90-Revised (SCL-90-R) (Derogatis, 2002; Almeida, 2006) was used. This self-report questionnaire is used to evaluate a range of current psychological symptoms and distress providing a Global Score index (GSI), which is a measure of the overall psychological distress and nine primary symptom dimensions (interpersonal sensitivity, depression, anxiety, hostility, phobic anxiety, paranoid ideation and psychoticism).

Sociodemographic and substance use data were collected through a questionnaire that, besides sociodemographic information, included items 10, 11 and 12 from the Alcohol Use Questionnaire (AUQ) (Townshend and Duka, 2002), assessing speed of drinking (average number of drinks consumed per hour), number of times getting drunk in the past 6 months, and percentage (average) of times getting drunk during drinking episodes.

Additionally, a diary of alcohol ingestion, questions about consumption of alcohol and other psychoactive substances (type of substance, frequency of consumption, etc.) and the Portuguese version of the Alcohol Use Disorder Identification Test (AUDIT) (Cunha, 2002) were administered. Total AUDIT score reflects the subject’s level of risk due to harmful alcohol intake: scores in the range of 8–19 reveal hazardous drinking, while scores of 20 or above warrant further diagnostic evaluation for alcohol dependence (Babor et al., 2001).

Impulsivity was assessed through the Barratt Impulsiveness Scale 11 (BIS11) (Patton et al., 1995). BIS is a self-report questionnaire intended to evaluate personality and behavioral aspects of impulsiveness providing a full-scale score plus second and first order subscores reflecting subtraits of impulsiveness. The Portuguese version was used (Cruz and Barbosa, 2012, Unpublished). Likewise, the Edinburg Handedness Inventory (Oldfield, 1971) was used to assess participants’ laterality.

### Procedure

All the participants (regardless of whether they had been pre-classified as BDs or AACs) underwent the same clinical, neuropsychological and neuroimaging assessment protocol. Prior to the MRI assessment, participants were asked to abstain from BD during the three preceding days, consuming drugs and alcohol 12 h before the scanning and to avoid smoking and drinking tea or coffee for at least 3 h in advance. All participants gave written informed consent after the procedure had been carefully explained and received a financial stipend for their participation. The research was conducted in accordance to the ethical principles for medical research involving human subjects of the World Medical Association present in the Declaration of Helsinki (Williams, 2008). The Bioethics Committee of the University of Minho approved the protocol.

### Magnetic Resonance Image Acquisition

The neuroimaging assessment was conducted with clinically approved Siemens Magneton TrioTim 3T MRI scanner (Siemens Medical Solutions, Erlangen, Germany) equipped with a 32-channel receive-only head coil. Sagittal high-resolution 3D T1 weighted anatomical images were acquired using a magnetization prepared rapid acquisition gradient echo (MPRAGE) sequence with the following parameters: repetition time (TR) = 2700 ms, echo time (TE) = 2.33 ms, flip angle (FA) = 7°, 192 slices with 0.8 mm thickness, in-plane resolution = 1 × 1 mm^2^, and 256 mm field of view (FoV).

### Image Processing

Before running the postprocessing protocol, all MRI scans were visually controlled to discard for critical head motion or brain lesions. All the images were normalized to the ICBM 152 average SPM template in Montreal Neurological Institute (MNI) space. Data was processed using SPM12 pipeline and statistical tools (Wellcome Trust Centre for Neuroimaging, University College London, United Kingdom^1^) executed in Matlab R2015a (MathWorks, Natick, MA, United States) with the VBM module. VBM is an automated processing technique applied to the entire brain allowing the characterization of shape and neuroanatomical configuration of different brains. Local composition of brain tissues is compared based in a voxelwise approach (Ashburner and Friston, 2000; Mechelli et al., 2005). Images were segmented into GM, white matter and cerebrospinal fluid using an extension of the standard unified segmentation model in SPM12. White and GM segmentations were co-registered across participants using the DARTEL algorithm (Diffeomorphic Anatomical Registration Through Exponentiated Lie Algebra; Ashburner, 2007; Ashburner and Friston, 2009) and smoothed with a 8 mm FWHM Gaussian filter to reduce errors from between-subject variability in local anatomy and to improve the normality of the data. For the purpose of this study only GM segmentations were analyzed.

### Region of Interest Definition (ROI)

**FIGURE 1:**
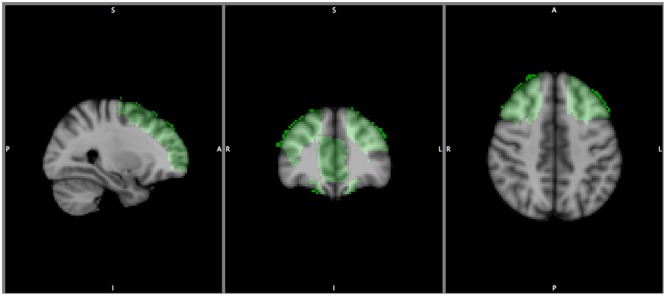
Represents the areas of the inhibitory circuitry.

For this purpose, a review on the prefrontal-striatal network underlying self-regulatory mechanisms involved in the addiction circuitry (Crews and Boettiger, 2009; Bava and Tapert, 2010; Koob and Volkow, 2010; Goldstein and Volkow, 2011; Koob, 2011; Bari and Robbins, 2013) was performed. The identified brain regions were the superior, middle and inferior frontal gyri, the frontal superior orbital gyrus, the anterior cingulum, the caudate nucleus and the nucleus accumbens. Therefore, a mask was generated with the WFUpickatlas toolbox version 3.0.5b^2^ based on the Talairach Daemon database running on MatLab R2015a (MathWorks, Natick, MA, United States) that included both cortical and subcortical areas of these brain regions, known to be involved in the inhibitory circuitry (see **Figure 1**).

### Statistical Analysis

For statistics, two-way analysis of variance was performed. Gender and Group were included as between subject factors and age was used as a covariate. Total intracranial volume of each subject was included in the statistical model. For statistical threshold criteria significant results were considered after Monte Carlo correction for multiple comparisons *p* < 0.05. The correction was determined over 1000 Monte Carlo simulations using AlphaSim tool, distributed with REST toolkit (Song et al., 2011)^3^ and mask set to the corresponding ROI previously generated. Anatomical labeling was obtained using the anatomical automatic labeling atlas (AAL), (Tzourio-Mazoyer et al., 2002).

In addition, Pearson correlations (with bootstrap corrections, 5000 iterations and 95% confidence interval) were performed to analyze the relationship between GM densities and both alcohol-related measures only in the BD group: number of times of binge drinking per month, number of months with BD pattern, grams of alcohol consumed per week, speed of drinking (grams/h during BD episodes), AUDIT scores, and BIS scores: total score, and subscales: attention, cognitive instability, motor control, perseverance, cognitive complexity and self-control. Additionally Pearson correlations between GM densities and the BIS scale and subscales scores were also calculated for both groups.

## Results

### ROI-Based Analysis

**FIGURE 2:**
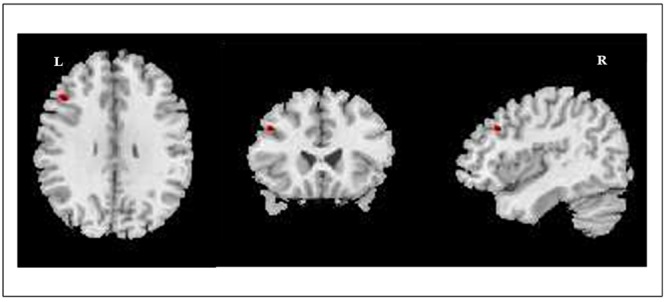
Image illustrates the regions where significant differences in GM densities (peak-level) were observed between BDs and AACs. BDs > AACs. Left middle frontal gyrus, MNI coordinates: –45, 24, 33; *K* = 315, *p* < 0.0001. Voxel size: 1.5 mm^3^.

Increased GM densities in the left middle frontal gyrus were observed in the BDs (MNI coordinates: -45, 24, 33; *K* = 315, *z* = 3.98, *p* < 0.0001 uncorrected; AlphaSim correction, *p* < 0.05, cluster size > 29), when compared to the AACs. **Figure 2** illustrates the regions where significant differences in peak-level densities were observed between BDs and AACs.

A group-by-gender interaction effect was observed in the left middle frontal gyrus (MNI coordinates: -42, 51, 15; *K* = 81, *z* = 3.40, *p* < 0.001 uncorrected; AlphaSim correction, *p* < 0.05, cluster size > 29). *Post hoc* tests revealed that BDs males displayed higher GM densities than AACs males in the left middle frontal gyrus (MNI coordinates: -44,26,33; *K* = 333, *z* = 3.86, *p* < 0.001 uncorrected); and BDs females displayed higher GM densities in the left middle frontal gyrus (MNI coordinates: -38, 58, 20; *K* = 40, *z* = 3.44, *p* < 0.001 uncorrected) when compared to AACs females.

No group differences were observed for white matter and gray matter densities between BDs and AACs (see Supplementary Table 1).

### Correlations

**FIGURE 3:**
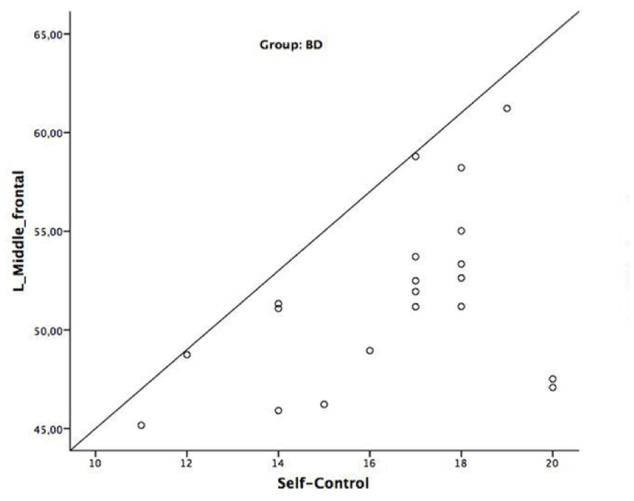
Image shows the positive associations between brain data (GM densities) and BIS (self-control) scores in the BD group.

Pearson correlations within the BD group revealed positive associations between the left middle frontal gyrus GM densities and the self-control subscale of the BIS (*r* = 0.45, *p* < 0.05 – see **Figure 3**). No significant correlations between GM densities and the BIS sub-factor self-control were observed in the AAC group. Finally, no significant correlations between GM densities and alcohol-related measures were observed in the BD group (see Supplementary Table 2).

## Discussion

In this VBM study, we used a ROI-based analysis of brain regions involved in the inhibitory circuitry and self-regulatory processes in a group of 20 BDs and 16 AACs. Overall we found increased GM densities in the left middle frontal gyrus of BDs when compared with their AACs counterparts. Additionally, we explored the associations between GM densities in the left middle frontal gyrus and BIS total scale and subscales scores. We found positive correlations between GM densities in the left middle frontal gyrus and scores in the BIS subscale self-control only in the BD group, that were not observed in the AAC group. Furthermore, no associations between GM densities and alcohol-related measures were observed in the BD group.

Increased GM densities in the middle frontal gyrus are consistent with previous studies (Squeglia et al., 2012; Doallo et al., 2014). In particular, thicker frontal areas were observed in female BDs (age range: 16–19 years old) in comparison with non-BDs females (Squeglia et al., 2012). Additionally, Doallo et al. (2014) found higher GM densities in the DLPFC of young BDs (age range: 20–24), with similar ages to our BD group (age range: 18–23), when compared to light consumers. These authors interpreted their findings as a consequence of high alcohol intake during important developmental periods such as adolescence and early adulthood. However, they also highlighted that these abnormalities could also be considered as a risk factor for heavy substance use (i.e., due to diminished efficiency in information processing and problem solving abilities, in addition to decreased ability in weighting risks vs. benefits), rather than a consequence of BD (Squeglia et al., 2012; Doallo et al., 2014).

Nevertheless, structural magnetic neuroimaging studies have produced inconsistent results regarding the effect of alcohol exposure in the volume and gray matter density of the frontal cortex, with studies showing no differences of decreased volumes or density in the alcohol consumers. In fact, Wilson et al. (2015) and Gropper et al. (2016) reported a deleterious effect of alcohol exposure in the ventral diencephalon, middle temporal gyrus and hippocampus, yet no significant effects on the frontal and parietal cortices. Other contradictory evidence was documented by a study using Orientation Dispersion Imaging, a method that assesses microstructural features directly related to neuronal morphology (Morris et al., 2017). In this study, the authors documented diminished dendritic complexity and organization in the DLPFC in a BD cohort (mean age: 22 years), despite no significant correlation between these measures and alcohol use severity was observed, suggesting that these neuronal abnormalities in BDs were not possibly modulated by the high alcohol intake. Longitudinal studies have also documented decreased volume and cortical thickness of the middle frontal gyrus (Wilson et al., 2015; Squeglia et al., 2017) in young adolescents (age range: 12–18) prior to alcohol use onset, which was further associated with alcohol initiation and BD. These results suggested that premorbid characteristics such as a delay of GM growth, which is a part of the typical normative neurodevelopmental process, could be associated with the BD onset (Squeglia et al., 2014, 2017; Wilson et al., 2015).

Such inconsistencies among studies are likely due to the use of different methods to perform the morphometric analysis of brain structure and volume or density (e.g., Luciana et al., 2013), as well as different age groups and participants with different patterns of consumption. While some of the studies analyzed cortical thickness (Luciana et al., 2013; Squeglia et al., 2017), others evaluated volumes or densities (Doallo et al., 2014), which limit the generalization of the findings, as these measures are not directly comparable. In fact, volume seems to be more closely related to surface area than to cortical thickness. Surface area and cortical thickness fluctuate along the course of brain development but not necessarily following the same direction or rate of variance than volume or densities (Winkler et al., 2009, 2010; Tamnes et al., 2017). Finally, different age ranges are associated with distinct neurodevelopment periods and can therefore represent an additional confounding factor. Overall, our findings showed increased GM densities in the left middle frontal gyrus in BD (age range: 18–23), which are in line with previous studies using participants with the same age interval (Doallo et al., 2014). Moreover, no differences in the total gray and white matter volumes between the BDs and the AAC group were observed, which excluded the impact of factors that could interfere with brain morphometry such as oedema or dehydration.

While our study cannot account for understanding these prefrontal brain abnormalities as a risk factor or as due to the neurotoxic effects of alcohol consumption, the morphologic changes in regions associated with self-regulatory processes that we observed in our BD group, could be related with two different hypotheses. The first hypothesis suggests that our result could be related with a delayed or an abnormal timing of the GM growth. In fact, and as it has been suggested by longitudinal studies, a reduction of gray matter was associated to a delay of gray matter growth in a prospective study of BDs before alcohol initiation (Squeglia et al., 2014, 2017; Wilson et al., 2015). Abnormalities in the middle frontal gyrus have been frequently associated to difficulties in regulating behavior when facing failure and undercontrol, and prospectively predicting substance use (age range: 9–12 years) (Heitzeg et al., 2014). In fact, a positive association between GM densities in the left middle frontal gyrus and scores in the self-control subscale of the BIS was also found in our BD group, which is consistent with others (Cho et al., 2013). A second hypothesis is that the abnormalities in the left middle frontal gyrus could be secondary to alcohol misuse, in accordance with other evidence (Crews and Boettiger, 2009; Bava and Tapert, 2010; Koob, 2011).

Nevertheless, from this study design, we cannot extrapolate about the causes and/or consequences of the BD behavior, as the cross sectional nature of the study is an important limitation. Other limitations include the need of bigger sample as necessary in order to increase the statistical power. The method of analysis (VBM) is an additional constraint. VBM is a fully automated method, as manual segmentation methods are considered the gold standard for structural neuroimaging studies. Finally, we could have had an additional control group including light or regular drinkers, in order to compare them with BDs and with the AACs. Future studies should take the advantage of longitudinal designs with more than two follow up assessments and the combination of morphometric, genetics and behavioral measures in order to disentangle whether structural abnormalities reflect vulnerability factors or consequences of high alcohol consumption.

## Conclusion

This study suggests frontal GM abnormalities in BDs college students, which is likely to impact self-regulatory processes. The pattern of increased regional GM density suggests that developmental factors may contribute to brain alterations in BDs.

## Author Contributions

SS wrote the manuscript, collected data, preprocessed the data, carried out the statistical analysis, and helped with subject’s recruitment and assessment. AS coordinated data acquisition, preprocessing and statistical analysis and collaborated in manuscript writing. PM helped with data preprocessing. OG collaborated in manuscript writing and AC designed the study, coordinated subject’s recruitment and assessment and data acquisition, and collaborated in manuscript writing. All authors read and approved the final manuscript.

## Conflict of Interest Statement

The authors declare that the research was conducted in the absence of any commercial or financial relationships that could be construed as a potential conflict of interest.

## Footnotes

1 http://www.fil.ion.ucl.ac.uk/spm/

2 http://www.ansir.wfubmc.edu

3 http://resting-fmri.sourceforge.net/

## Abbreviations

AAC: alcohol-abstinent control
AACs: alcohol-abstinent controls
AUDIT: Alcohol Use Disorder Identification Test
BD: binge drinking
BDs: binge drinkers
BIS: Barratt Impulsiveness Scale
DLPFC: dorsolateral prefrontal cortex
GM: gray matter
ROI: regions of interest
VBM: voxel-based morphometry
