# Supplementary material for: Gray Matter Abnormalities in the Inhibitory Circuitry of Young Binge Drinkers: A Voxel-Based Morphometry Study
Source: Front Psychol. 2017 Sep 13;8:1567. doi: 10.3389/fpsyg.2017.01567 (PMC5604066; doi:10.3389/fpsyg.2017.01567)
Supplement: Supplementary file 1 [file Data_Sheet_1.DOCX]

**Supplemental material:**

**Table 1. Gray and White matter Differences between BDs and AACs**

|  | **BDs** | **AACs** | ***t (34)*** | ***p*** |
| --- | --- | --- | --- | --- |
|  | **n = 20** | **n = 16** |  |  |
|  | **Mean (SD)** | **Mean (SD)** |  |  |
| Total Gray Matter (l) | 0.792 (0.070) | 0.755 (0.072) | -1.56 | 0.13 |
| Total White Matter (l) | 0.482 (0.065) | 0.459 (0.055) | -1.13 | 0.27 |

The p-values reported are for 2-tailed independent samples t-tests

**Table 2. Correlations between BIS Scores and Gray Matter Densities in the BD and AAC groups**

|  | **Left Middle Frontal** | | | |
| --- | --- | --- | --- | --- |
|  | **BDs** | | **AACs** | |
|  | **Pearson (*r*)** | ***p*** | **Pearson (*r*)** | ***p*** |
| BIS (Total Score) | 0.02 | 0.92 | 0.38 | 0.15 |
| BIS (Attention) | -0.14 | 0.57 | 0.26 | 0.33 |
| BIS (Cognitive Instability) | -0.18 | 0.44 | 0.15 | 0.58 |
| BIS (Motor) | -0.06 | 0.82 | 0.15 | 0.57 |
| BIS (Perseverance) | 0.36 | 0.12 | 0.28 | 0.30 |
| BIS (Self-Control) | 0.45 | 0.049* | 0.25 | 0.35 |
| BIS (Cognitive Complexity) | -0.32 | 0.17 | 0.09 | 0.73 |

*Correlation is significant at the 0.05 level (2-tailed).
